# *Mannheimia haemolytica* Negatively Affects Bovine Herpesvirus Type 1.1 Replication Capacity In Vitro

**DOI:** 10.3390/microorganisms10112158

**Published:** 2022-10-31

**Authors:** Caitlyn A. Cowick, Brynnan P. Russ, Anna R. Bales, Bindu Nanduri, Florencia Meyer

**Affiliations:** 1 Department of Biochemistry & Molecular Biology, Entomology & Plant Pathology, Mississippi State University, 408 Dorman Hall, 32 Creelman St., Box 9655, Starkville, MS 39762, USA; 2 Department of Comparative Biomedical Sciences, College of Veterinary Medicine, Mississippi State University, Starkville, MS 39762, USA

**Keywords:** bovine respiratory disease (BRD), co-infection, bovine herpesvirus, *Mannheimia haemolytica*, *Pasteurella multocida*

## Abstract

Bovine Respiratory Disease (BRD) is a multifactorial condition affecting cattle worldwide resulting in high rates of morbidity and mortality. The disease can be triggered by Bovine Herpesvirus-1 (BoHV-1) infection, stress, and the subsequent proliferation and lung colonization by commensal bacteria such as *Mannheimia haemolytica*, ultimately inducing severe pneumonic inflammation. Due to its polymicrobial nature, the study of BRD microbes requires co-infection models. While several past studies have mostly focused on the effects of co-infection on host gene expression, we focused on the relationship between BRD pathogens during co-infection, specifically on *M. haemolytica’s* effect on BoHV-1 replication. This study shows that *M. haemolytica* negatively impacts BoHV-1 replication in a dose-dependent manner in different in vitro models. The negative effect was observed at very low bacterial doses while increasing the viral dose counteracted this effect. Viral suppression was also dependent on the time at which each microbe was introduced to the cell culture. While acidification of the culture medium did not grossly affect cell viability, it significantly inhibited viral replication. We conclude that *M. haemolytica* and BoHV-1 interaction is dose and time-sensitive, wherein *M. haemolytica* proliferation induces significant viral suppression when the viral replication program is not fully established.

## 1. Introduction

Bovine respiratory disease (BRD) is a polymicrobial condition in which viruses, bacteria, environmental stressors and management practices converge to negatively impact the health of cattle [1]. BRD is one of the most important health issues facing cattle, accounting for high morbidity and mortality with a large economic impact on a global scale [2,3,4]. After varied stressors such as weaning, transportation, social re-organization, metabolic/dietary shifts or extreme weather, viruses such as bovine herpesvirus type 1 (BoHV-1) or Bovine respiratory syncytial virus (BRSV) infect and replicate in the respiratory epithelium, inducing host immunosuppression and allowing resident opportunistic resident bacteria such as *Mannheimia haemolytica* or *Pasteurella multocida* to proliferate and translocate to the lung [5,6,7,8,9]. The simultaneous viral [10,11,12] and bacterial [13] infections contribute to rapidly deteriorate the health status of animals.

Common viruses associated with BRD are BoHV-1 [1,14], BRSV [10], bovine parainfluenza 3 [12,15], bovine viral diarrhea virus [11], and somewhat less consistently bovine coronavirus and bovine influenza D virus [16,17,18,19]. BoHV-1 is an enveloped double-stranded DNA virus, a member of the alphaherpesvirinae subfamily. Subtype 1 (BoHV-1.1) is typically isolated from bovine respiratory infections but can also infect the genital tract of cows and bulls [20]. It is transmitted mainly by cough droplets, direct contact of oral/nasal biofluids, genital secretions, or vertically from mother to fetus [1,20,21]. Once in the host, BoHV-1 is a tenant for life and remains in a latent state in the trigeminal ganglia and tonsils [22,23], reactivating from latency after stressful stimuli such as those listed earlier [5].

*M. haemolytica* and *P. multocida* are commensal, Gram-negative coccobacilli [24,25,26]. There are several strains of *M. haemolytica* found in the upper respiratory tract nasal flora of cattle, but serotype S1 is predominantly associated with most cases of BRD [27]. After replicating in the upper respiratory mucosa, *M. haemolytica* serotype S1 can translocate to the lung leading to fibrinous nectrotizing bronchopneumonia [27,28]. During active infection, *M. haemolytica* produces leukotoxin (LKT) during the logarithmic growth phase, which targets leukocytes within the host and triggers an apoptotic response resulting in cell death and inflammation [29,30,31]. This bacterium also contains lipopolysaccharide (LPS) layer in its cell wall that serves as an endotoxin, contributing to the production of inflammatory cytokines and apoptosis of host cells [32].

Due to the polymicrobial etiology of BRD, many studies have used co-infection experimental designs to answer pathogenesis-related questions. Early in vivo work showed that certain BRD microbe combinations exacerbated respiratory signs and disease, with stress playing a central role [1,33,34,35]. For instance, BoHV-1 and *M. haemolytica* co-infection exhibited a synergistic relationship in animal challenge studies, where infection with BoHV-1 triggered the expression of pro-inflammatory cytokines and Toll-like receptor-dependent signaling which in turn promoted *M. haemolytica*’s replication, infection of mononuclear cells, and increased adherence to the bronchial epithelium [5,35,36]. In addition, leukocyte recruitment and inflammatory cytokines was hypothesized to have contributed to worsen the effects of the leukotoxin in the lung [36,37]. Similar studies have shown that epithelial cultures pre-infected with BRSV promoted the adherence of *P. multocida* [38], particularly in cultures derived from the lower respiratory epithelium [39]. However, studies conducted with other BRD microbe combinations have shown that not all co-infections lead to enhanced morbidity. Lin et al., shows that introducing *Histophilus somni* before BRSV in vitro can suppress BRSV replication, suggesting that the order in which microbes establish an infection affects the overall outcome of infection [40]. Similarly, *P. multocida* and *M. haemolytica* were shown to mutually inhibit each other when forming a joint biofilm on respiratory epithelium cell cultures [41]. However, *P. multocida* cooperated with *H. somni* to enhance the formation of a biofilm [42]. Co-infection studies have focused on host gene expression after single/multiple pathogen infection and have shown that distinct transcriptional patterns are induced by different BRD agents [43,44,45,46], as well as by single versus dual-pathogen infections [37]. However, the concept of direct microbial interference on viral replication is particularly relevant to BRD because commensal bacteria exist at low levels when a viral infection is established. The current study focused on how BRD microbes affect each other in the context of co-infection. The objective was to characterize the impact of *M. haemolytica* and *P. multocida* on BoHV-1 replication.

## 2. Materials and Methods

### 2.1. Cell Culture

Madin-Darby Bovine Kidney (MDBK) (ATCC CCL-22) epithelial cells were grown in Dulbecco’s Modified Eagle Medium (DMEM) (Corning) (4.5 g/L glucose, L-glutamine, & sodium pyruvate), containing 5% gamma-irradiated Fetal Bovine Serum (FBS) (Egua Fetal, Atlas Biological) and 100 mg/mL of Penicillin and Streptomycin (P/S) (Corning Cellgro/Fisher) (hereto referred to as complete medium). Bovine Turbinate (BT) (ATCC CRL 13%) cells were grown in DMEM containing 10% gamma-irradiated FBS (EquaFetal, Atlas Biologicals) and 100 mg/mL of P/S. When cells were to be infected with live *M. haemolytica* or *P. multocida*, P/S was omitted. Cell cultures were maintained at 37 °C in the presence of 5% CO_2_.

To subculture cells, cultures were rinsed twice with phosphate-buffered saline (PBS) (137 mM NaCl; 2.7 mM KCl; 10 mM Na_2_HPO4; 1.8 mM KH_2_PO_4_; pH 7.4) and trypsinized using 0.25% trypsin (Corning) in PBS for 30 min at 37 °C in the presence of 5% CO_2_. Cells were stained using bromophenol blue and counted using a hemocytometer. In certain experiments the pH of medium was adjusted with 1 M HCl.

### 2.2. Viral and Bacterial Strains

BoHV-1 (Cooper strain) was used in all co-infections to infect MDBK or BT cells. Viral stocks were prepared in MDBK cells and stored at −80 °C in DMEM without antibiotics for use during co-infection of cells in the presence of bacteria.

*M. haemolytica* D153 serotype A1 strain and *P. multocida* 3480 were obtained from Dr. Nanduri’s lab, and cultured in Brain-Heart Infusion (BHI, SIGMA) broth at 37 °C, with shaking or streaked on BHI agar plates. Overnight single-colony bacterial cultures were diluted on the day of the experiment. Optical density (OD) at 600 nm was measured using a microplate reader (BioTek Synergy SLXFA).

### 2.3. Co-Infection of MDBK or BT Cells with BoHV-1 and M. haemolytica

MDBK or BT cells were seeded into multi-well tissue culture plates and incubated at 37 °C in the presence of 5% CO_2_ for 24 h. BoHV-1 was added at a multiplicity of infection (MOI) 1 or 5 viral particles per cell, unless otherwise noted. A single-colony overnight culture of *M. haemolytica* was serially diluted in DMEM in a range from 1:10 to 1:1,000,000,000 (referred to as dilutions 10^−1^ to 10^−9^ throughout the study) and added to bovine cultures either simultaneously or 2–4 h after BoHV-1 infection. The estimated cfu/mL for each dilution at the time of inoculation is provided in Table S1. Aliquots of supernatant or whole cells were collected at various hours post infection (hpi) and stored at −80 °C.

### 2.4. Viral Titration

Standard plaque assay was performed to quantify the number of infectious viral particles in the samples. MDBK cells were seeded into 6-well tissue culture plates (1 × 10^6^ cells per well) and incubated overnight. Wells were washed with PBS. Samples to be quantified were serially diluted in DMEM containing antibiotics without FBS and 1 mL of each serial dilution pipetted into a well. Plates were incubated for an hour at 37 °C in 5% CO_2_ incubator with recurrent gentle shaking, rinsed with PBS, followed by the addition of 1 mL of a 1:1 mixture of 1% agarose in PBS and 10% FBS DMEM. After overlay gelification, plates were returned to the CO_2_ incubator for an additional 48 h. Plates were fixed and stained with a 4% paraformaldehyde (PFA) and crystal violet solution. Removal of the agarose overlay allowed to visualize cells. Viral titer was calculated by counting the number of plaques, or small clearings on the cell monolayer, and expressed as plaque forming units per mL (pfu/mL).

### 2.5. MTT Cell Viability Assay

The MTT cell viability assay kit (Biotium) was used to test metabolic function of cells. Following manufacturer’s recommendations, MDBK cells were seeded into a 96-well tissue culture plate (6 × 10^3^ cells per well). After completion of an experiment, cells were washed with PBS and 10 µL of MTT solution was added to the wells, mixed gently and incubated for 4 h at 37 °C in a 5% CO_2_ atmosphere. 200 µL of dimethyl sulfoxide (DMSO) was added to the mixture to solubilize the tetrazolium salt that was produced by the metabolically active cells and absorbance was measured at 570 nm. Background absorbance was measured at 630 nm and subtracted from signal absorbance to yield normalized absorbance values.

### 2.6. Bromophenol Blue Exclusion Viability Assay

After completion of an experiment, cells were gently scraped from culture wells with a cell scraper and the contents of the well placed in 1.5 mL tubes. Tubes were spun down at 4000× *g* for 5 min and the supernatant discarded. Cellular pellets were gently washed in 1mL of PBS and spun down again for 5 min. Cellular pellets were resuspended in 1 mL of trypsin and incubated for 15 min to obtain a single cell suspension suitable for counting. In a separate tube, trypsinized cells were diluted 1:5 using 1% bromophenol blue in PBS. Dead cells appear blue due to a compromised cell membrane permeability, while live cells appear bright under the microscope due to the exclusion of the dye. The number of live/dead cells were counted in a hemocytometer and averaged (*n* = 4).

### 2.7. Spent Supernatant and LPS Assay

MDBK cells were seeded into 96-well tissue culture plates and incubated overnight at 37 °C in the presence of 5% CO_2_. An overnight culture of *M. haemolytica* was (a) diluted to 1:100 in fresh BHI broth or DMEM without P/S and incubated at 37 °C with shaking, or (b) diluted 1:100 in DMEM without P/S and incubated in the presence of MDBK cells at 37 °C for 8 h (log phase of growth). At 8 h, each culture was spun down at 12,000 rpm for 5 min and the supernatant was filtered using a syringe and 0.45 µm syringe filter. Half of the filtered supernatant was set aside, and the other half was placed in a boiling water bath for 10 min. Both samples (boiled and non-boiled) were then added directly to freshly prepared MDBK cells as a 1:10 dilution and allowed to incubate for 24 h at 37 °C in the presence of 5% CO_2_. For the LPS assay, fresh overnight monolayers were inoculated with various dilutions of *Escherichia coli* LPS (serotype EH100; 1.0 mg/mL, Enzo Life Sciences) and also incubated for for 24 h at 37 °C in the presence of 5% CO_2_. Plates were then washed twice with PBS and stained with 4% PFA and crystal violet solution.

### 2.8. Statistical Analysis

The sample standard deviation (SD) was used to calculate error bars for all graphs. Results are expressed as the mean +/− SD and are representative of three or more independent experiments. One tailed Student’s *t*-test with unequal variance was used to calculate statistical significance with alpha = 0.05 or 0.01. In all graphs, * indicates *p* ≤ 0.05 and ** indicates *p* ≤ 0.01.

## 3. Results

### 3.1. Bacterial Replication Negatively Impacts BoHV-1 Replication in Cultured Bovine Cells

**Figure 1:**
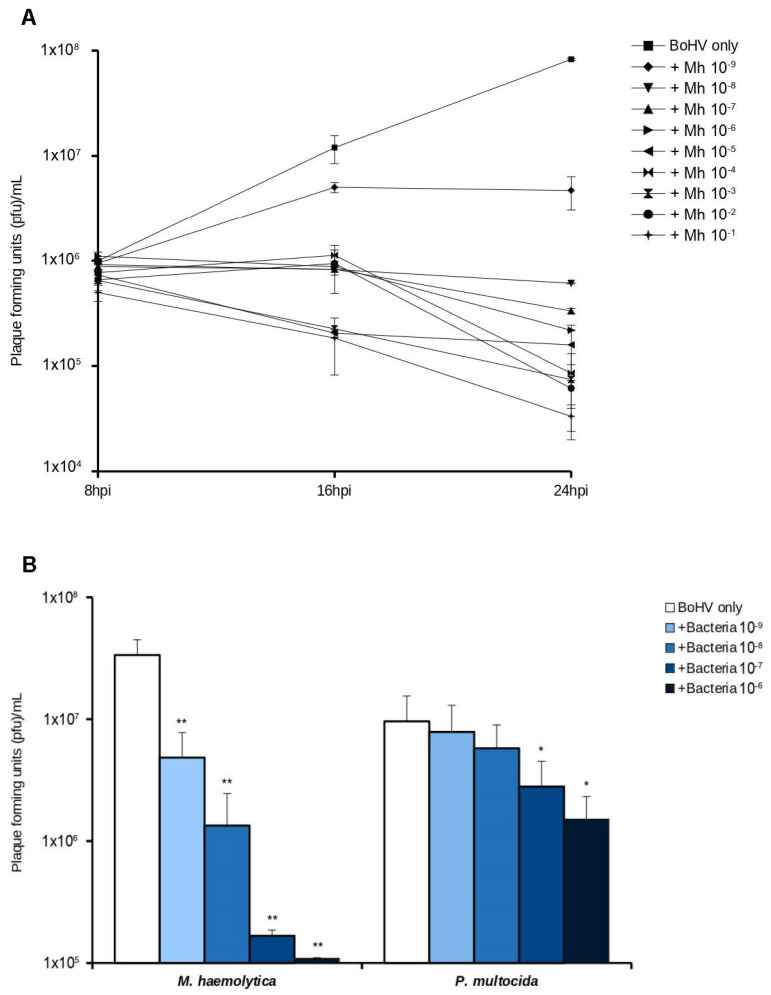
(**A**) Simultaneous co-infection of MDBK cells with BoHV-1 and *M. haemolytica*. MDBK cells were infected simultaneously with increasing doses of bacteria (dilutions 10^−6^–10^−9^) and BoHV-1 (MOI 1) for 24 h. Culture supernatants were collected at 8, 16, and 24 hpi and pfu/mL were quantified. (**B**) Viral quantification resulting from simultaneous co-infection with BoHV-1 (MOI 1) and *M. haemolytica* or *P. multocida* (dilution range 10^−6^ to 10^−9^). Significance levels between the means were tested by comparing against the “BoHV only” infection in each group (*, *p* < 0.05; **, *p* < 0.01).

Initial experiments were designed to study the effects of bacterial replication on the ability of BoHV-1 to establish an infection and replicate. After establishing that *M. haemolytica* could efficiently replicate in DMEM medium (Supplementary Figure S1), BoHV-1 (MOI 1) and increasing dilutions of *M. haemolytica* (10^−1^–10^−9^ from overnight cultures) were simultaneously inoculated into cultured MDBK cells. The replication of BoHV-1 in the presence of *M. haemolytica* was significantly reduced (*p* < 0.05) starting at 16 hpi and showed a greater reduction at 24 hpi (Figure 1A). The lowest bacterial dose (dilution 10^−9^) significantly inhibited BoHV-1 replication by ten times at 24 hpi compared to cells infected only with BoHV-1. The decrease in viral output was also statistically significant at all the higher *M. haemolytica* doses tested. Subsequent experiments were conducted only with the four lowest bacterial doses (dilutions 10^−6^–10^−9^). Viral titers at 24 hpi for the four lowest bacterial doses are shown in Figure 1B. A tabular format of the data presented as percent reduction in viral output (Table 1) further highlights an 85% reduction in the production of infectious virus at 24 hpi when *M. haemolytica* was co-inoculated at the lowest dose. Co-infection carried out with BoHV-1 and *P. mutocida* showed a similar pattern in which bacterial growth negatively affected BoHV-1 replication. However, for *P. multocida* a higher dose was needed to induce a significant decrease in viral output (dilution 10^−7^ in Figure 1B and Table 1). In addition the amount of bacteria added at 0 h was also higher (Supplementary Table S1), suggesting that *P. mutocida* may have milder effect on BoHV-1.1.

**Figure 2:**
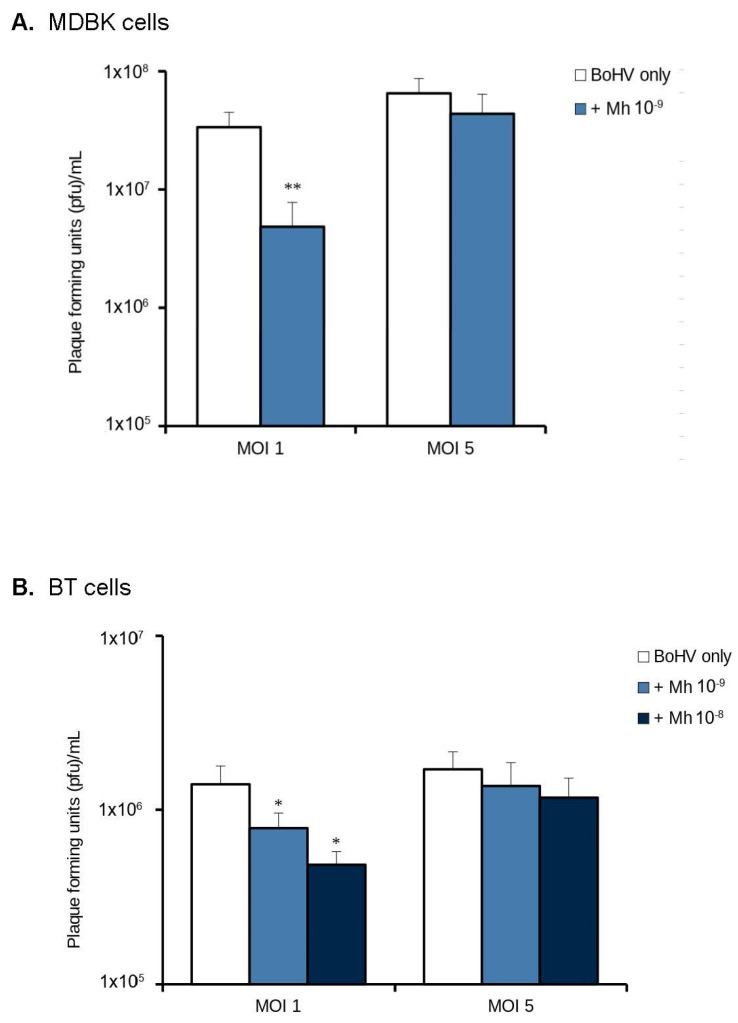
Higher viral dose counteracts *M. haemolytica’s* adverse effect. Increasing doses of *M. haemolytica* (dilutions 10^−9^ to 10^−6^) were added simultaneously with BoHV-1 at either MOI 1 or MOI 5 to MDBK panel (**A**) or BT cells panel (**B**). Viral replication was assessed by plaque assay (pfu/mL). Significance levels between the means were tested by comparing against the “BoHV only” infection in each group (*, *p* < 0.05; **, *p* < 0.01).

We next tested BoHV-1’s ability to overcome the suppression in replication induced by *M. haemolytica* by increasing the virus infectious dose (or multiplicity of infection, MOI). At MOI 5, the anti-viral effect induced by *M. haemolytica* was less pronounced and did not result in a significant decrease in viral replication at the lowest bacterial dose, dilution 10^−9^ (Figure 2A). We further confirmed these results using bovine turbinate (BT) cells in co-infection experiments. BT cells are derived from newborn bovine turbinate tissue located within the nasal passages and are routinely used in BRD research. BoHV-1 replication in BT cells was consistently about 10 times lower than in MDBK cells (Figure 2B). During co-infection with *M. haemolytica*, a similar effect in viral replication was observed (Figure 2B), where BoHV-1 replication (MOI 1) decreased significantly in the presence of low doses of *M. haemolytica*, and increasing the viral dose to MOI 5 counteracted the adverse effect. Collectively, these results suggest that *M. haemolytica*‘s replication negatively affects BoHV1′s replication program in both cell types, yet the negative effect can be overcome by a larger virus dose.

### 3.2. Timing of Bacterial Infection Influences the Anti-Viral Effect

**Figure 3:**
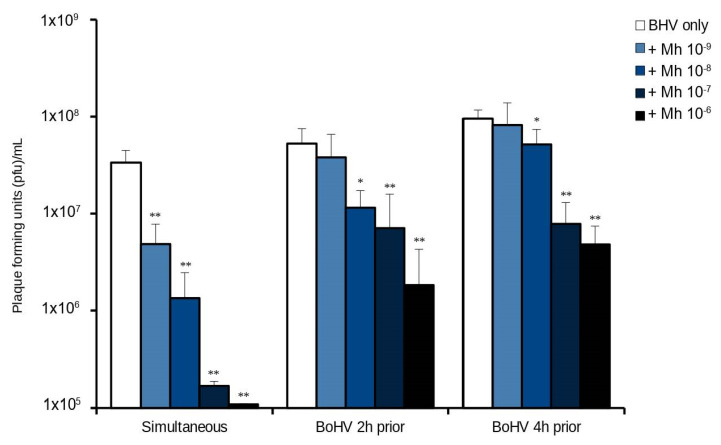
Timing of the BoHV-1—*M. haemolytica* co-infection is important. BoHV-1 (MOI 1) was added either simultaneously, 2 h or 4 h prior to adding increasing doses of *M. haemolytica* (dilutions 10^−6^–10^−9^). Viral replication was assessed by plaque assay (pfu/mL). Significance levels between the means were tested by comparing against the “BoHV only” infection in each group (*, *p* < 0.05; **, *p* < 0.01).

We next investigated whether *M. haemolytica*‘s replication would suppress viral replication if those cells were already infected with virus at the time of bacterial inoculation. MDBK cells were infected with BoHV-1 for 2 or 4 h prior to the addition of *M. haemolytica*. Contrary to the previous experiment, the lowest bacterial dose (dilution 10^−9^) no longer led to significant reduction of viral replication at 24 h (Figure 3). However, the reduction in viral replication was still significant at the 10^−8^ and higher tested bacterial doses when BoHV-1 was added 2 h (*p* = 0.015) or 4 h (*p* = 0.023) prior to *M. haemolytica.* Overall, this experiment illustrated that the longer time allowed for BoHV-1 to establish its replication program, a higher *M. haemolytica* dose was needed to significantly reduce viral output.

### 3.3. Cell Viability

**Figure 4:**
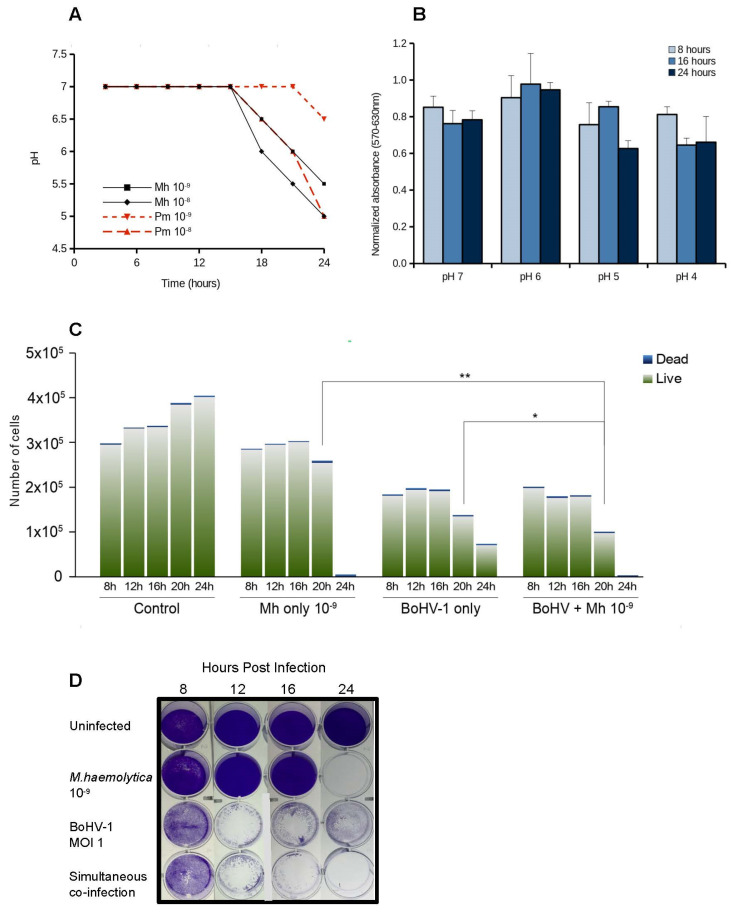
Acidification and MDBK cell viability. (**A**) Acidification during *M. haemolytica* and *P. multocida* growth in DMEM. (**B**) MDBK cell viability after culture at indicated pH. Cell viability was assessed by MTT assay. (**C**) Cell viability of MDBK cells infected with BoHV-1, *M. haemolytica,* or simultaneously with both agents was assessed at the indicated times by live/dead counts using bromophenol blue exclusion assay. Error bars are not shown due to the stacked nature of the plot. However, statistically significance between some samples’ means is indicated with asterisks (*, *p* < 0.05; **, *p* < 0.01). (**D**) MDBK cells were fixed and stained at the indicated times post infection with BoHV-1, *M. haemolytica,* or both to depict the viability of the cell monolayer.

We conducted an adherence and invasion assay [25] to confirm that *M. haemolytica* did not affect cell viability by invading cells (Supplementary Table S2). We next asked whether acidification caused by bacterial replication affected cell viability. We assessed the extent and timing of bacterial metabolic acidification, as well as cell viability at different pH. *M. haemolytica* and *P. multocida* were grown in DMEM (pH of 7) and statically incubated at 37 °C and 5% CO_2_. *M. haemolytica* seeded at the lowest concentration (10^−9^ dilution) did not begin media acidification until 16 h, after which the pH progressively decreased to about 5.5 at 24 h (Figure 4A). *P. multocida* exhibited a slower acidification rate at the lowest doses. We additionally assessed MDBK cell viability under this pH range using the MTT cell viability assay, which detects metabolically active cells. Figure 4B shows that MDBK cells remained viable over a period of 24 h in the 5–7 pH range, likely due to the buffering capacity of DMEM culture medium. These results indicated that medium acidification in this range did not impact cell viability.

We next assessed cell viability in the context of microbial co-infection. MDBK cells were infected with either BoHV-1 (MOI 1), *M. haemolytica* (10^−9^ dilution), or simultaneously co-infected with both pathogens. Uninfected cells served as a control. At five different time points live/dead cells were quantified (Figure 4C). Cells infected with *M. haemolytica* showed no significant decline in live cell counts by 20 h. However, no viable cells remained at 24 h. Wells containing cells infected only with virus had sustained level of viable cells at 8, 12 and 16 hpi but were overall lower than control or cells infected with bacteria (statistically significant, asterisks not shown). At 20 hpi cells showed a significant decline in viability that also continued to 24 hpi (statistically significant, not shown). Cells co-infected with both pathogens followed similar viability as virus-only infected cells up to 16 hpi, but progressed to significant cellular destruction at 20 hpi. Cell viability in co-infected wells at 20 hpi was significantly lower than that of cells infected with either microbe alone. A visual assessment of crystal-violet stained cells after infection confirmed the similarity in cell viability timeline between BoHV-1 only and co-infection of BoHV and *M. haemolytica* (Figure 4D, bottom two rows). By 24 h the collective effects of either bacterial or bacterial+viral infection appeared to completely impair cell viability. Collectively these experiments suggested that during co-infection the main driver of cellular death, starting at 20 hpi, was BoHV-1 infection or a combinatorial effect of both pathogens.

**Figure 5:**
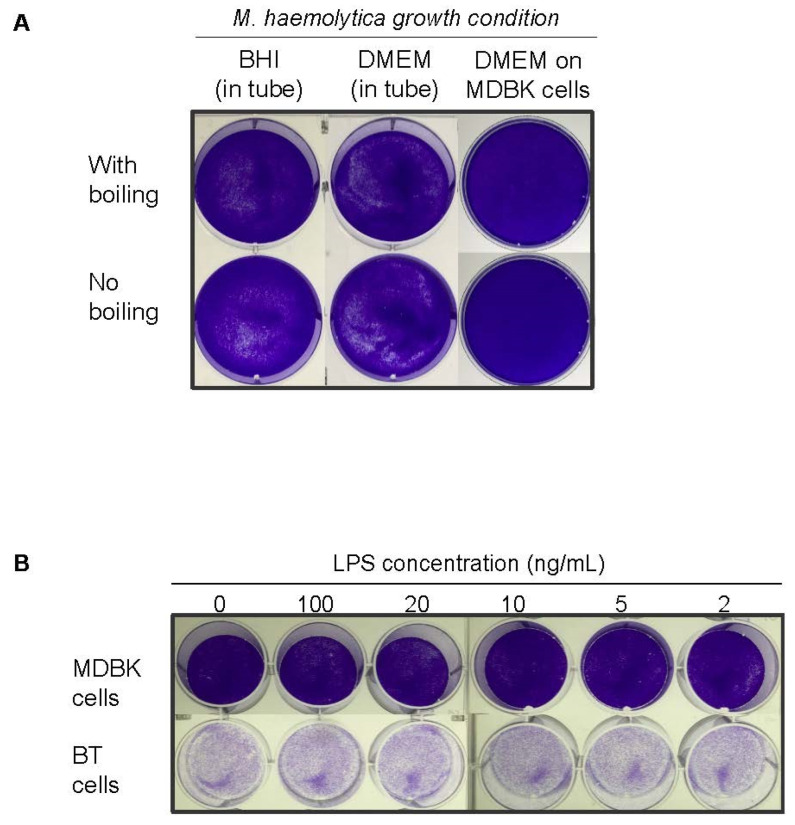
*M. haemolytica* may not produce soluble factors cytolytic to MDBK or BT cells. (**A**) An overnight culture of *M. haemolytica* was diluted 1:100 and incubated either in a tube with fresh BHI or fresh DMEM, or in DMEM in the presence of MDBK cells. After 8 h, filtered supernatants were boiled or left untreated and applied to fresh cell monolayers. Cells were washed, fixed and stained 24 h after treatment. (**B**) LPS was directly applied to fresh MDBK and BT cell monolayers at the indicated concentrations. Cells were washed, fixed and stained 24 h after treatment.

We further tested whether soluble bacterial factors were cytotoxic, thus indirectly reducing viral output. *M. haemolytica* was grown in tubes containing BHI or DMEM, or in DMEM in the presence of MDBK cells to about mid-log phase (8 h), based on LKT expression kinetics [47]. Culture supernatants were filtered to remove bacterial cells and subjected to boiling to inactivate soluble factors. Filtered supernatants were then applied to fresh MDBK cell monolayer and incubated 24 h. The results showed no difference in the appearance of the monolayer in any of these scenarios (Figure 5A). To test whether bacterial LPS may be affecting cell viability, concentrated *Escherichia coli* LPS was inoculated into fresh MDBK and BT cell monolayers. Figure 5B shows that the appearance of LPS-treated cell monolayers did not differ from untreated controls, even when the highest dose of LPS was applied. Overall, these experiments suggested that bacterial toxins are not affecting cellular viability under our experimental conditions.

### 3.4. Low pH Negatively Affects BoHV-1 Replication

**Figure 6:**
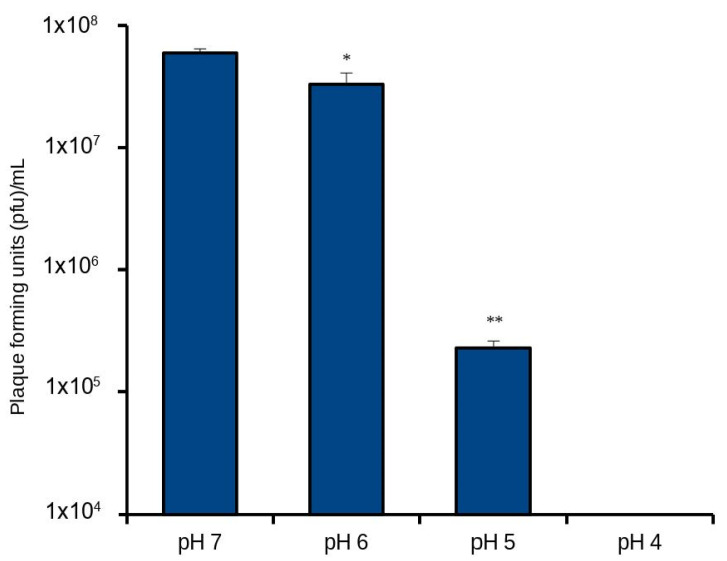
Impact of pH on BoHV-1 replication. BoHV1 (MOI 1) was allowed to infect MSBK cells with an increasingly acidic medium. The applied pH range was 7–4. Viral titers were quantified at 24 hpi by plaque assay. The limit of detection for this assay is 10^3^ pfu/mL. Significance levels (*, *p* < 0.05; **, *p* < 0.01).

Results shown in Figure 4 and Figure 5 suggested that pH in the culture medium did not directly affect cell viability. We therefore asked whether pH could affect the viral cycle. In this experiment we tested the effect of an acidic environment on BoHV-1 replication. We infected MDBK cells with BoHV-1 (MOI 1) in a pH range of 7–2. The results shown in Figure 6 indicate that a pH of 5 had a drastic impact in BoHV-1’s replication capacity, decreasing viral output by about 2 orders of magnitude. Below pH 4, BoHV-1 completely lost the ability to replicate.

## 4. Discussion

Joint microbial colonization of the respiratory tract epithelium is the hallmark of bovine respiratory disease and constitutes the main obstacle to the development of measures to reduce BRD incidence. Studying joint microbial infection is therefore one important facet for understanding how microbes may interact as they colonize the same tissues. This in vitro study reports a direct effect of *M. haemolytica’s* metabolism on BoHV-1 that does not appear to involve the host cell. Overall, our results showed that (a) the dose of each infecting pathogen was important for the outcome of the infection (Figure 1 and Figure 2). The antiviral effect caused by the lowest bacterial dose dissipated if the viral MOI increased from 1 to 5 in both cell types; (b) the time of infection dictated the efficiency of viral output, giving BoHV-1 an advantage when it was allowed to establish an infection 2–4 h ahead of *M. haemolytica* (Figure 3); and (c) BoHV-1 could not replicate below pH of 5 in vitro (Figure 6), which is the same acidification range caused by *M. haemolytica*’s metabolism.

The reduced viral replication reported in this study is comparable to the reported reduction in viral titers of BRSV in co-infection studies with *Histophilus somni* [40]. Lin and colleagues described a 30-fold reduction in BRSV titers in infected bovine alveolar type 2 (BAT-2) cells after treating cells with *H. somni* concentrated culture supernatant. This study also noted an upregulation in the expression of cellular antiviral genes, such as SIG15, MX or viperin, in cultured cells. These changes in host gene expression and viral output were attributed to a yet unknown *H. somni* factor secreted into the culture medium [40]. In contrast, our present study found no evidence of *M. haemolytica* secretion of soluble factors that might be indirectly reducing viral output by compromising cell integrity (Figure 5).

The observed reduction in viral titers in BT cells supported our initial experiments in MDBK cells. Given the respiratory origin of BT cells, these results could have in vivo relevance as *M. haemolytica* is already present when BoHV-1 initially infects a host or reactivates from latency at the epithelial surface. *M haemolytica* grows to high density in liquid culture (about 10^10^ cfu/mL). A 10 ^−9^ dilution of *M. haemolytica* culture used in our experiments would be in the 10 CFU range (Supplementary Table S1). This low number of cells is likely similar or even smaller than a low-level presence of *M. haemolytica* in the respiratory mucosa. A study quantifying microbial load in nasopharyngeal swabs of pre-weaned Holstein calves observed a somewhat consistent total bacterial load of 10^5^ bacterial cells/swab (as measured by 16S rRNA gene copies via quantitative PCR) [8]. The mean relative abundance of *Mannheimia* and *Pasteurella* (genus level) was 4–20% depending on the study, and <1–7% at the species level (*M. haemolytica and P. multocida*) [6,8,48,49]. Another recent study of bacterial carriage in healthy cattle used PCR to estimate *M. haemolytica* and *P. multocida* densities in nasal passages found the mean colony count/mL to be 2–4 log_10_ genome copies of *M. haemolytica* and 4–6 log_10_ genome copies of *P. multocida* [50]. When looking at our results through the lens of these quantification studies, we conclude that the low bacterial doses used for our experiments are comparable or lower to what could be found in vivo. Thus, it is plausible that a microbial interaction of this nature could take place on respiratory surfaces. If BoHV-1’s lytic infection program was established 2 or 4 h before *M. haemolytica* began to replicate locally, the viral output could be about 10 times higher (Figure 3) in that small area of respiratory epithelium. On the other hand, if both microbes were to begin replicating on the mucosa simultaneously, the acidification brought about by bacterial metabolism would suppress viral replication significantly. The observed dose-dependent interplay between BoHV-1 and *M. haemolytica* or *P. multocida*, and the fact that these are commensal respiratory bacteria, suggests the order and extent of microbial colonization is directly relevant to viral replication success: the establishment of a poductive BoHV-1 viral infection may be dampened or not depending on the preexisting local concentration of *M. haemolytica.* This raises the challenging idea that carriage of a certain load of *M. haemolytica* may be beneficial to suppress or keep viral replication under control. However, appropriate animal studies are needed to test this hypothesis about microbial interaction in vivo. Importantly, not all commensal microbes may have the same effect on BoHV-1 output, since we observed that *P. multocida* required at least a 100-fold higher dose to negatively affect viral replication in vitro. Further work is needed to address the significance of this observation.

Two of *M. haemolytica*‘s toxins responsible for much of the cytopathology observed in vivo are LKT and LPS [29,32]. Our results suggest that *M. haemolytica* may not produce soluble virulence factors in vitro that are cytotoxic to MDBK or BT cells (Figure 5). LKT was reported to be synthesized in the log phase when cultured in a modified BHI in vitro [47]. However, under the experimental conditions in this study *M. haemolytica* did not appear to produce a cytotoxin. The receptor for LKT on its main cell target, bovine macrophages and neutrophils, was shown to be integrin beta 2 (or CD18) [51,52,53]. It is therefore also possible that MDBK and BT cells do not sufficiently express this surface receptor. Further studies are needed to establish the the range of surface receptor expression patterns in MDBK cells.

Viral entry of human herpesvirus-1 (HSV-1) and that of BoHV-1 are known to be dependent on a low-pH-mediated endocytosis pathway [54,55]. A mildly acidic pH of 6 in the endosome is needed for a conformational change to take place in the envelope glycoprotein B, allowing viral fusion with the host cell plasma membrane and successful entry [56]. Our results supported the conclusions of these studies, showing that a pH below 5 has an inhibitory effect on the virus’s ability to replicate. A pH of 5 is similar to the acidification range caused by *M. haemolytica*’s replication and as such it may be effective in limiting BoHV-1 infection of neighboring epithelial cells. Therefore, while our results suggest that pH did not adversely affect healthy host cells in the tested time frame, the reduced pH had an adverse effect on viral replication. 

The BRD field has long recognized that the imbalance of microbial populations is a strong contributing factor to disease. How dysbiosis is triggered or triggers BRD is not clear and is the focus of several current studies [57,58]. In recognizing the value of maintaining a diverse respiratory microbiome, novel approaches explore how to target *M. haemolytica* colonization while avoiding metaphylactic antimicrobial treatments [59]. Our results suggest a potential beneficial role for a low level carriage of *M. haemolytica*. Future work on the relationship between these and other pathogens that jointly persist and proliferate on respiratory tissues will bring light to new aspects of this complex disease. 

## Figures and Tables

**Table 1:** Percent reduction in viral replication when in co-infection. Data used in Figure 1B is presented as a % reduction in viral replication with respect to the control sample (cells singly infected with BoHV-1.1). MDBK cells were infected with BoHV-1.1 only (MOI 1) or with BoHV-1.1 and *M. haemolytica* or *P. multocida* diluted as shown. Supernatants were assayed for viral infectius particles at 24 h. % reduction = 100 − (bacteria + virus pfu/mL counts)/(virus pfu/mL counts) × 100. Highlighted (bold) is the bacterial dilution at which the reduction was significant (see also Figure 1B).

	% Reduction in Viral Output (with Respect to BoHV-1.1)	
↓ Microbes Involved (Dilution)	*+ Mannheimia haemolytica*	*+ Pasteurella multocida*
BoHV-1.1	--	--
BoHV-1.1 + Bacteria (10^−9^)	**85.60%**	18.1%
BoHV-1.1 + Bacteria (10^−8^)	96.0%	39.9%
BoHV-1.1 + Bacteria (10^−7^)	99.5%	**70.8%**
BoHV-1.1 + Bacteria (10^−6^)	99.7%	84.3%

## Data Availability

The data supporting the conclusions of this article are included within the article and its figures. No large datasets were generated or analyzed during the current study.

## Supplementary Materials

The following supporting information can be downloaded at: https://www.mdpi.com/article/10.3390/microorganisms10112158/s1, Figure S1: *M. haemolytica* exhibits normal growth in DMEM medium. MDBK cells were seeded into wells containing DMEM plus 5% FBS but containing no antibiotics, and incubated overnight at 37 °C and a 5% CO_2_ atmosphere. Single-colony *M. haemolytica* were grown overnight in BHI and diluted 1:100 into wells containing cultured MDBK that had been seeded the night before. Wells were either uninfected (no infection), infected with *M. haemolytica*, or infected with both *M. haemolytica* and Bovine Herpesvirus type 1 (MOI 1). Optical Density was monitored ar regular intervals; Table S1: Colony forming units for dilutions made from single-colony overnight cultures of *M. haemolytica* and *P. multocida*. 10-fold serial dilutions were plated on BHI agar plates in triplicates, and colony forming units per mL (cfu/mL) were counted after 24 h of incubation at 37 °C; Table S2: Adherence and invasion assay. MDBK cells were infected with M. haemolytica (1:10) and incubated for 2, 4, and 6 h. Antibiotics (penicillin and streptomycin, P/S) was then added to the cultures for 1 h, followed by cell lysis and plating on BHI agar.
