# Supplementary material for: Mannheimia haemolytica Negatively Affects Bovine Herpesvirus Type 1.1 Replication Capacity In Vitro
Source: Microorganisms. 2022 Oct 31;10(11):2158. doi: 10.3390/microorganisms10112158 (PMC9697469; doi:10.3390/microorganisms10112158)
Supplement: Supplementary file 1 [file microorganisms-10-02158-s001.zip › microorganisms-2003551-supplementary.pdf]

Supplementary Material

Mannheimia haemolytica negatively affects Bovine Herpesvirus type 1.1 replication capacity in vitro.

Caitlyn A. Cowick, Brynnan P. Russ, Anna Bales, Bindu Nanduri and Florencia Meyer

Table S1. Colony forming units for dilutions made from single-colony overnight cultures of *M. haemolytica* and *P. multocida*. 10-fold serial dilutions were plated on BHI agar plates in triplicates, and colony forming units per mL (cfu/mL) were counted after 24 hours of incubation at 37ºC.

	Average cfu/mL	
Dilution from o/n culture	*Mannheimia haemolytica*	*Pasteurella multocida*
1×10^{-6}	2.23×10^{2}	2.2×10^{4}
1×10^{-7}	3.67×10^{1}	4.7×10^{3}
1×10^{-8}	2.60×10^{1}	1.5×10^{3}
1×10^{-9}	1.07×10^{1}	8.4×10^{2}
1×10^{-10}	9.00×10^{0}	2.0×10^{1}
1×10^{-11}	2.00×10^{0}	9.0×10^{0}

Table S2. Adherence and invasion assay. MDBK cells were infected with *M. haemolytica* (1:10) and incubated for 2, 4, and 6 hours. Antibiotics (penicillin and streptomycin, P/S) was then added to the cultures for 1 hour, followed by cell lysis and plating on BHI agar.

	Average cfu/mL	
Time (hours)	No P/S added	P/S added
2	1×10^{7}	0
4	8×10^{7}	0
6	3×10^{8}	0

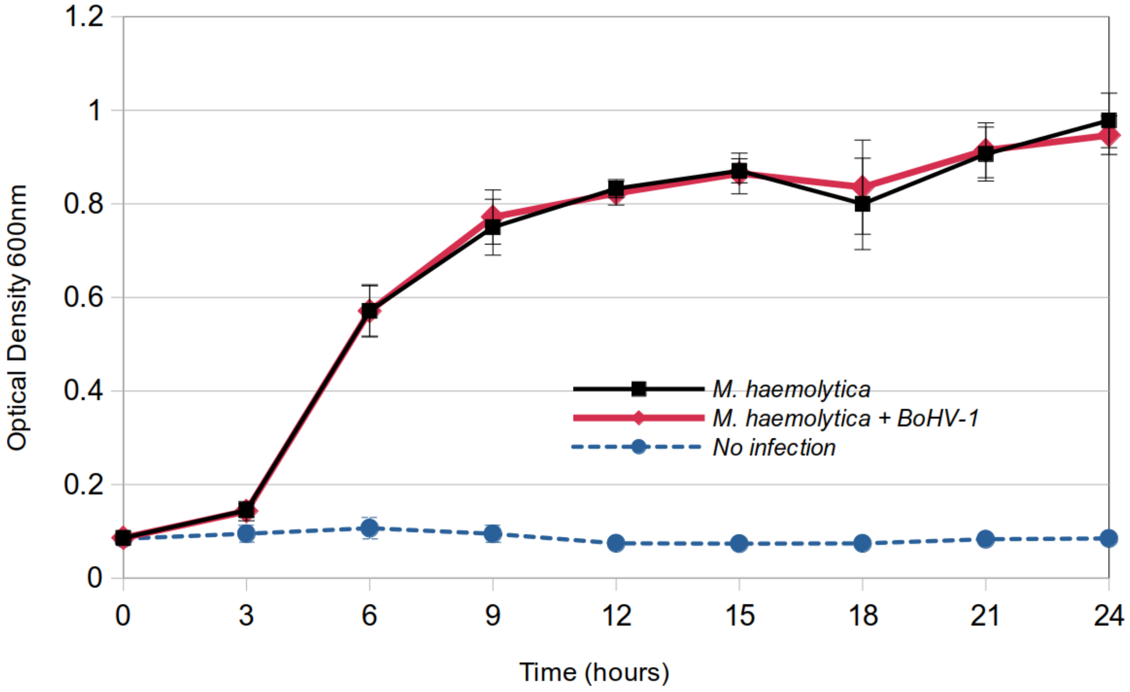

Figure S1. *M. haemolytica* exhibits normal growth in DMEM medium. MDBK cells were seeded into wells containing DMEM plus 5% FBS but containing no antibiotics, and incubated overnight at 37°C and a 5% CO_2 atmosphere. Single-colony *M. haemolytica* were grown overnight in BHI and diluted 1:100 into wells containing cultured MDBK that had been seeded the night before. Wells were either uninfected (no infection), infected with *M. haemolytica,* or infected with both *M. haemolytica* and Bovine Herpesvirus type 1 (MOI 1). Optical Density was monitored ar regular intervals.
